# A case of pyoderma gangrenosum around the urethral meatus aggravated by COVID‐19 infection and further worsened due to the development of pyogenic osteomyelitis 8 years after urostomy

**DOI:** 10.1002/ccr3.7501

**Published:** 2023-06-13

**Authors:** Makoto Kondo, Yoshiaki Matsushima, Takehisa Nakanishi, Koji Habe, Keiichi Yamanaka

**Affiliations:** ^1^ Department of Dermatology Mie University Graduate School of Medicine Tsu Japan

**Keywords:** coronavirus‐19, inflammatory cytokines, pyoderma gangrenosum, pyogenic osteomyelitis, urostomy

## Abstract

After the infection with COVID‐19, pyoderma gangrenosum worsened and further led to necrosis following pyogenic osteomyelitis. Infection is a major exacerbating factor in pyoderma gangrenosum.

## Graphical abstract

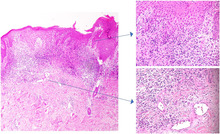

Pyoderma gangrenosum (PG) is a form of neutrophilic dermatosis, commonly developing at sites of injury due to the typical pathergy phenomenon.^1^, ^2^ We encountered a 73‐year‐old male patient who had a history of bladder and right renal cancer for which he had undergone a urostomy. Apart from this, he had no other significant medical history. The patient had been using a pouch over his urethral meatus for 8 years without trouble. He presented to our department with the chief complaint of multiple small ulcers around the urethral meatus that had started to merge. An ulcer had formed over the urethral meatus, with raised margins resembling an embankment (Figure 1A).

**FIGURE 1:**
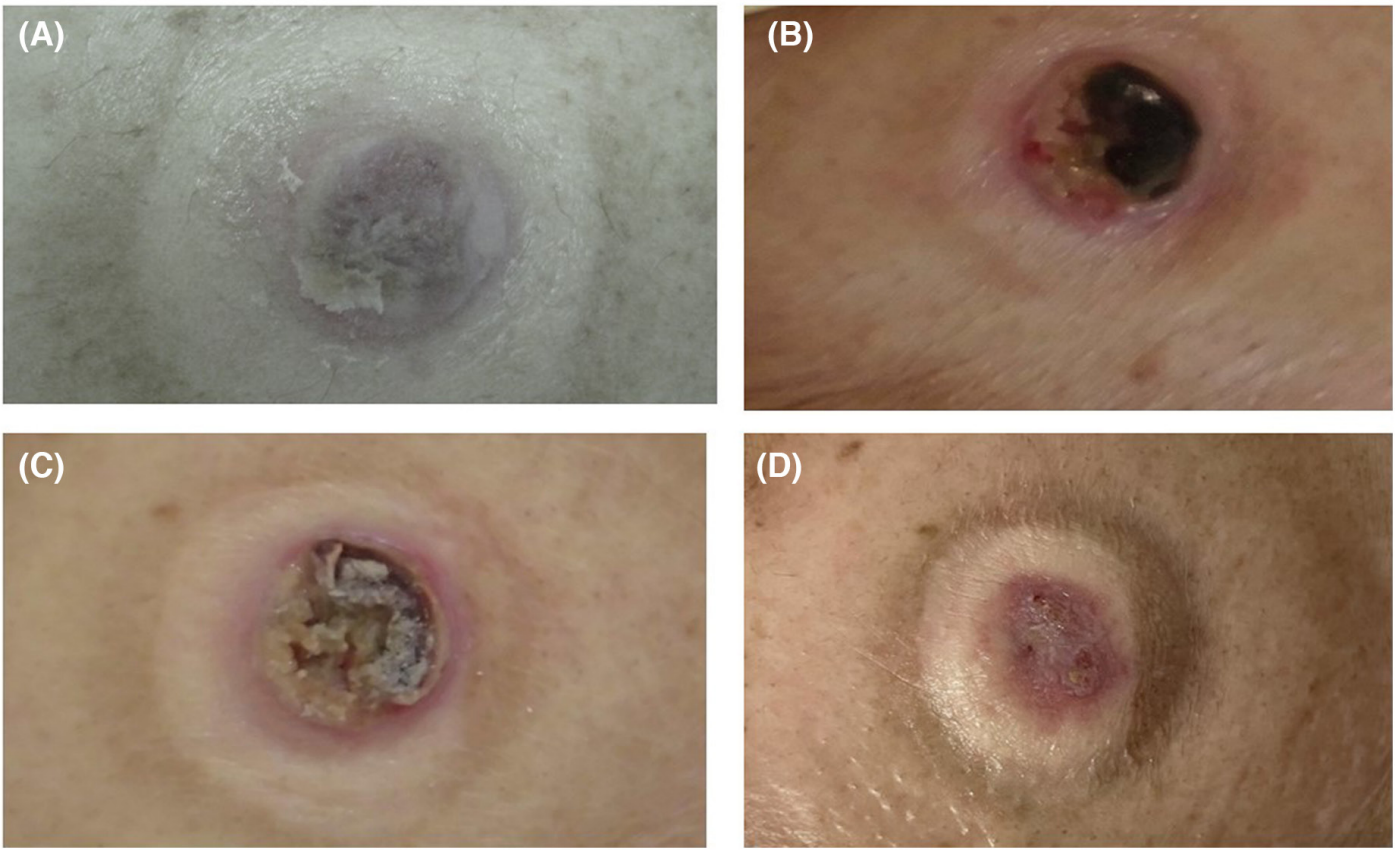
(A) Fused small ulcers formed the plaque of shallow ulcers with embankment‐like ridges at the margins. (B) The ulcer had deepened with bleeding and erythematous margins. (C) The ulcer around the urethral meatus showed necrosis and collapse. (D) The erythema around the ulcer had disappeared and epithelialization had progressed.

However, the patient did not exhibit symptoms of dysuria or hematuria. Although *Proteus mirabilis* was detected in the ulcer, there were no conspicuous signs of infection. Skin biopsy taken from the raised margins of the ulcer revealed a zonal lymphocytic infiltrate and edema beneath the thickened epidermis, a finding consistent with PG (Figure 2). No associated symptoms were observed such as periorbital inflammation or intraorbital lesions, which can sometimes accompany PG. A month subsequent to these observations, after testing positive for COVID‐19, the patient was hospitalized. Clinical laboratory tests showed elevated liver enzymes (AST: 59 IU/L, ALT: 23 IU/L), inflammatory markers (WBC: 17,600/μL with 90.4% neutrophils, CRP: 16.51 mg/dL), indicators of renal function (BUN: 60.0 mg/dL, Cre: 1.83 mg/dL), and a marker of muscle breakdown (CPK: 2711 IU/L). The ulceration around the urethral meatus had worsened, causing bleeding, and erythema was observed in the surrounding area (Figure 1B). Two weeks into hospitalization and treatment, the patient started experiencing back pain and was diagnosed with pyogenic osteomyelitis via an MRI scan. Concurrently, the ulcer over the urethral meatus had become necrotic (Figure 1C). For treatment, the patient was administered ceftriaxone (2 g/day) and levofloxacin (500 mg/day) for a month. The patient's condition improved without any sequelae. Furthermore, the ulceration at the urethral meatus spontaneously resolved along with the improvement of the infections, without the need for topical medications or oral steroids (Figure 1D).

**FIGURE 2:**
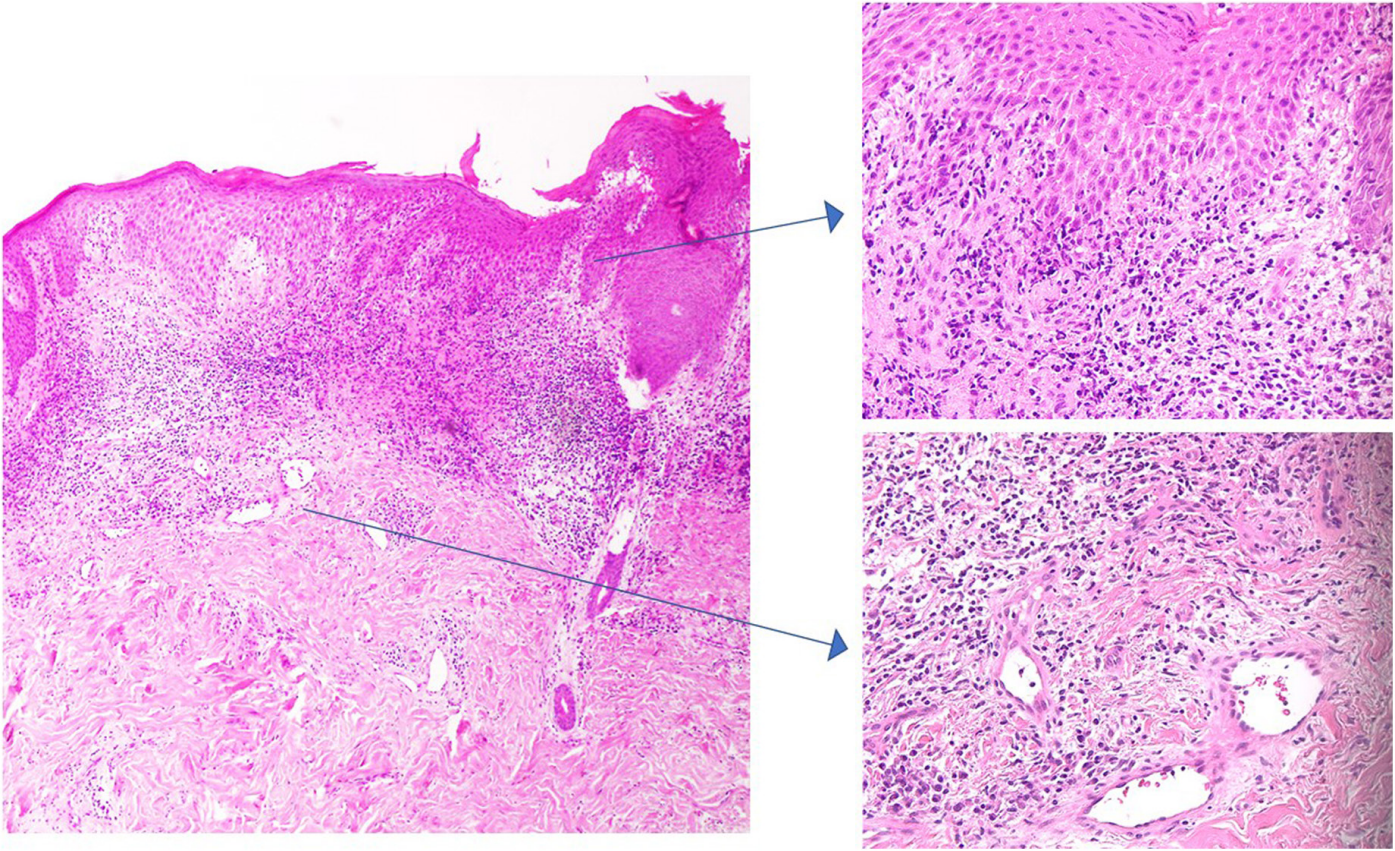
Edematous changes and inflammatory cell infiltration, primarily neutrophils, are observed from the dermis to the subcutaneous tissue. Mild vasculitis is also present. The overall image (Hematoxylin and eosin stain magnification: ×40). zoomed‐in image (Hematoxylin and eosin stain magnification: ×200).

Our case presents a rare occurrence of pyoderma gangrenosum (PG) worsening following a COVID‐19 infection, leading to the development of pyogenic osteomyelitis and necrosis of the ulcer. The patient experienced a fever and was hospitalized due to the coronavirus infection, although the blood sample findings were atypical for viral infections. We speculate that PG had been slowly progressing since the initial appearance of small ulcers around the urethral meatus, and the ulcer rapidly deteriorated after the COVID‐19 infection. This suggests that the PG around the urethral meatus was exacerbated by the release of inflammatory cytokines during the COVID‐19 infection, which strongly stimulated the type 1 immune system. Previous reports have documented cases of PG emerging after COVID‐19 infection or vaccination.^3^ The condition was further aggravated by the presence of pyogenic osteomyelitis. Physicians should be aware of this rare phenomenon when treating patients with unexplained ulcers around the urethral meatus.

## AUTHOR CONTRIBUTIONS

**Makoto Kondo:** Conceptualization; data curation; investigation; writing – original draft. **Yoshiaki Matsushima:** Data curation. **Takehisa Nakanishi:** Data curation. **Koji Habe:** Writing – review and editing. **Keiichi Yamanaka:** Project administration; writing – review and editing.

## FUNDING INFORMATION

The authors did not receive any financial support for this study.

## CONFLICT OF INTEREST STATEMENT

The authors declare no competing interests.

## ETHICAL APPROVAL

This study is a medical case report, and ethical approval was not required for this study in accordance with local and national guidelines.

## CONSENT

Written informed consent was obtained from the patient for publication of the details of their medical case and any accompanying images.

## Data Availability

All data that support the findings of this study are included in this article.
